# Protocol for developing a core outcome set for male infertility research: an international consensus development study

**DOI:** 10.1093/hropen/hoac014

**Published:** 2022-03-16

**Authors:** Michael P Rimmer, Ruth A Howie, Richard A Anderson, Christopher L R Barratt, Kurt T Barnhart, Yusuf Beebeejaun, Ricardo Pimenta Bertolla, Siladitya Bhattacharya, Lars Björndahl, Pietro Bortoletto, Robert E Brannigan, Astrid E P Cantineau, Ettore Caroppo, Barbara L Collura, Kevin Coward, Michael L Eisenberg, Christian De Geyter, Dimitrios G Goulis, Ralf R Henkel, Vu N A Ho, Alayman F Hussein, Carin Huyser, Jozef H Kadijk, Mohan S Kamath, Shadi Khashaba, Yoshitomo Kobori, Julia Kopeika, Tansu Kucuk, Saturnino Luján, Thabo Christopher Matsaseng, Raj S Mathur, Kevin McEleny, Rod T Mitchell, Ben W Mol, Alfred M Murage, Ernest H Y Ng, Allan Pacey, Antti H Perheentupa, Stefan Du Plessis, Nathalie Rives, Ippokratis Sarris, Peter N Schlegel, Majid Shabbir, Maciej Śmiechowski, Venkatesh Subramanian, Sesh K Sunkara, Basil C Tarlarzis, Frank Tüttelmann, Andy Vail, Madelon van Wely, Mónica H Vazquez-Levin, Lan N Vuong, Alex Y Wang, Rui Wang, Armand Zini, Cindy M Farquhar, Craig Niederberger, James M N Duffy

**Affiliations:** MRC Centre for Reproductive Health, Queens Medical Research Institute, University of Edinburgh, Edinburgh, UK; Edinburgh Fertility Centre, Simpsons Centre for Reproductive Health, Royal Infirmary of Edinburgh, Edinburgh, UK; MRC Centre for Reproductive Health, Queens Medical Research Institute, University of Edinburgh, Edinburgh, UK; Edinburgh Fertility Centre, Simpsons Centre for Reproductive Health, Royal Infirmary of Edinburgh, Edinburgh, UK; Reproductive Medicine Research Group, University of Dundee, Ninewells Hospital and Medical School, Dundee, UK; Department of Obstetrics and Gynaecology, University of Pennsylvania School of Medicine, Philadelphia, PA, USA; King’s Fertility, The Fetal Medicine Research Unit, King’s College London, London, UK; Division of Urology, Department of Surgery, Universidade Federal de Sao Paulo, Sao Paulo, Brazil; University of Aberdeen, Aberdeen, UK; ANOVA—Karolinska University Hospital and Karolinska Institutet, Stockholm, Sweden; The Ronald O. Perelman and Claudia Cohen Centre for Reproductive Medicine, Weill Cornell Medicine, New York, NY, USA; Northwestern University, Feinberg School of Medicine, Chicago, IL, USA; University of Groningen, University Medical Centre, Groningen, Centre of Reproductive Medicine, Groningen, Netherlands; Asl Bari, Reproductive Unit and Andrology Clinic, Conversano (Ba), Italy; RESOLVE: The National Infertility Association, McLean, VA, USA; Nuffield Department of Women’s and Reproductive Health, University of Oxford, Oxford, UK; Women’s Centre, John Radcliffe Hospital, Headington, Oxford, UK; Stanford University, Stanford, CA, USA; Reproductive Medicine and Gynaecological Endocrinology (RME), University Hospital, University of Basel, Basel, Switzerland; Units of Human Reproduction and Reproductive Endocrinology, 1st Department of Obstetrics and Gynaecology, Medical School, Aristotle University of Thessaloniki, Thessaloniki, Greece; Department of Digestion, Metabolism and Reproduction, Imperial College London, London, UK; IVFMD, My Duc Hospital, HOPE Research Centre, My Duc Hospital, Ho Chi Minh City, Vietnam; Minia University, Minia, Egypt; Reproductive Biology Laboratory, Department of Obstetrics and Gynaecology, University of Pretoria, Steve Biko Academic Hospital, Pretoria, South Africa; Freya—Dutch Patient Association for Infertility, Gorinchem, The Netherlands; Christian Medical College, Vellore, India; University of New South Wales, Sydney, Australia; IVF Australia, Sydney, Australia; Dokkyo Medical University Saitama Medical Center, Mibu, Japan; Guy’s and St Thomas Hospital, London, UK; Acibadem Maslak Hospital, Istanbul, Turkey; Urology Department, Hospital Universitari i Politècnic La Fe, Valencia, Spain; Stellenbosch University, Stellenbosch, Western Cape, South Africa; Tygerberg Academic Hospital, Cape Town, South Africa; Manchester University Foundation Trust, Manchester Academic Health Sciences Centre, Manchester, UK; Newcastle Fertility, The Newcastle upon Tyne Hospitals NHS Trust, Newcastle upon Tyne, UK; MRC Centre for Reproductive Health, Queens Medical Research Institute, University of Edinburgh, Edinburgh, UK; University of Aberdeen, Aberdeen, UK; Department of Obstetrics and Gynaecology, Monash University, Clayton, Australia; Harley Street Fertility Centre, Nairobi, Kenya; Department of Obstetrics and Gynaecology, The University of Hong Kong, Hong Kong Special Administrative Region, China; Department of Oncology and Metabolism, University of Sheffield, Sheffield, UK; Department of Obstetrics and Gynaecology, University of Turku and Turku University Hospital, Turku, Finland; College of Medicine, Mohammed Bin Rashid University of Medicine and Health Sciences, Dubai, UAE; Medical Physiology, Stellenbosch University, Tygerberg, South Africa; Rouen University Hospital, Biology of Reproduction-CECOS Laboratory, Rouen, France; King’s Fertility, The Fetal Medicine Research Unit, King’s College London, London, UK; Faculty of Life Sciences and Medicine, King’s College London, London, UK; The Ronald O. Perelman and Claudia Cohen Centre for Reproductive Medicine, Weill Cornell Medicine, New York, NY, USA; Guy’s and St Thomas Hospital, London, UK; Association for Infertility Treatment and Adoption Support “Our Stork”, Warsaw, Poland; King’s Fertility, The Fetal Medicine Research Unit, King’s College London, London, UK; Faculty of Life Sciences and Medicine, King’s College London, London, UK; Units of Human Reproduction and Reproductive Endocrinology, 1st Department of Obstetrics and Gynaecology, Medical School, Aristotle University of Thessaloniki, Thessaloniki, Greece; Institute of Reproductive Genetics, University of Münster, Münster, Germany; Centre for Biostatistics, University of Manchester, Manchester Academic Health Science Centre, Manchester, UK; Netherlands Satellite of the Cochrane Gynaecology and Fertility Group, Centre for Reproductive Medicine, Amsterdam, Netherlands; Reproduction & Development Research Institute, Amsterdam University Medical Centre, Amsterdam, Netherlands; Laboratorio de Estudios de Interacción Celular en Reproducción y Cáncer, Instituto de Biología y Medicina Experimental (IBYME), Consejo Nacional de Investigaciones Científicas y Técnicas de Argentina (CONICET), Fundación IBYME (FIBYME), Buenos Aires, Argentina; Department of Obstetrics and Gynaecology, University of Medicine and Pharmacy at Ho Chi Minh City, Ho Chi Minh City, Vietnam; HOPE Research Centre, My Duc Hospital, Ho Chi Minh City, Vietnam; Faculty of Health, University of Technology Sydney, Ultimo, Australia; Department of Obstetrics and Gynaecology, Monash University, Melbourne, Australia; Division of Urology, Department of Surgery, McGill University, Montreal, Quebec, Canada; Cochrane Gynaecology and Fertility Group, University of Auckland, Auckland, New Zealand; Department of Obstetrics and Gynaecology, University of Auckland, Auckland, New Zealand; Department of Urology, University of Illinois at Chicago, Chicago, IL, USA; Department of Bioengineering, University of Illinois at Chicago College of Engineering, Chicago, IL, USA; King’s Fertility, The Fetal Medicine Research Unit, King’s College London, London, UK

**Keywords:** core outcome set, clinical practice guidelines, consensus study, fertility, male fertility, modified Delphi method, randomized controlled trials, reproductive healthcare, reproduction, systematic review

## Abstract

**STUDY QUESTION:**

We aim to develop, disseminate and implement a minimum data set, known as a core outcome set, for future male infertility research.

**WHAT IS KNOWN ALREADY:**

Research into male infertility can be challenging to design, conduct and report. Evidence from randomized trials can be difficult to interpret and of limited ability to inform clinical practice for numerous reasons. These may include complex issues, such as variation in outcome measures and outcome reporting bias, as well as failure to consider the perspectives of men and their partners with lived experience of fertility problems. Previously, the Core Outcome Measure for Infertility Trials (COMMIT) initiative, an international consortium of researchers, healthcare professionals and people with fertility problems, has developed a core outcome set for general infertility research. Now, a bespoke core outcome set for male infertility is required to address the unique challenges pertinent to male infertility research.

**STUDY DESIGN, SIZE, DURATION:**

Stakeholders, including healthcare professionals, allied healthcare professionals, scientists, researchers and people with fertility problems, will be invited to participate. Formal consensus science methods will be used, including the modified Delphi method, modified Nominal Group Technique and the National Institutes of Health’s consensus development conference.

**PARTICIPANTS/MATERIALS, SETTING, METHODS:**

An international steering group, including the relevant stakeholders outlined above, has been established to guide the development of this core outcome set. Possible core outcomes will be identified by undertaking a systematic review of randomized controlled trials evaluating potential treatments for male factor infertility. These outcomes will be entered into a modified Delphi method. Repeated reflection and re-scoring should promote convergence towards consensus outcomes, which will be prioritized during a consensus development meeting to identify a final core outcome set. We will establish standardized definitions and recommend high-quality measurement instruments for individual core outcomes.

**STUDY FUNDING/COMPETING INTEREST(S):**

This work has been supported by the Urology Foundation small project award, 2021. C.L.R.B. is the recipient of a BMGF grant and received consultancy fees from Exscentia and Exceed sperm testing, paid to the University of Dundee and speaking fees or honoraria paid personally by Ferring, Copper Surgical and RBMO. S.B. received royalties from Cambridge University Press, Speaker honoraria for Obstetrical and Gynaecological Society of Singapore, Merk SMART Masterclass and Merk FERRING Forum, paid to the University of Aberdeen. Payment for leadership roles within NHS Grampian, previously paid to self, now paid to University of Aberdeen. An Honorarium is received as Editor in Chief of *Human Reproduction Open.* M.L.E. is an advisor to the companies Hannah and Ro. B.W.M. received an investigator grant from the NHMRC, No: GNT1176437 is a paid consultant for ObsEva and has received research funding from Ferring and Merck. R.R.H. received royalties from Elsevier for a book, consultancy fees from Glyciome, and presentation fees from GryNumber Health and Aytu Bioscience. Aytu Bioscience also funded MiOXYS systems and sensors. Attendance at Fertility 2020 and Roadshow South Africa by Ralf Henkel was funded by LogixX Pharma Ltd. R.R.H. is also Editor in Chief of *Andrologia* and has been an employee of LogixX Pharma Ltd. since 2020. M.S.K. is an associate editor with *Human Reproduction Open*. K.Mc.E. received an honoraria for lectures from Bayer and Pharmasure in 2019 and payment for an ESHRE grant review in 2019. His attendance at ESHRE 2019 and AUA 2019 was sponsored by Pharmasure and Bayer, respectively. The remaining authors declare no competing interests.

**TRIAL REGISTRATION NUMBER:**

Core Outcome Measures in Effectiveness Trials (COMET) initiative registration No: 1586. Available at www.comet-initiative.org/Studies/Details/1586.

**TRIAL REGISTRATION DATE:**

N/A.

**DATE OF FIRST PATIENT’S ENROLMENT:**

N/A.

WHAT DOES THIS MEAN FOR PATIENTS? Male infertility affects millions of men world-wide, and many different treatments have been proposed for this. How effective these treatments are can only be truly understood if clinical trials report the same outcomes, which are measured and defined in the same way. The protocol described here sets out the process by which we will develop a multinational, multiprofessional driven ‘core outcome set’ for future male infertility research.

Currently, there is no agreed consensus on what outcomes clinical trials should collect and report when evaluating treatments for male infertility. This means that when new trials are published to evaluate a treatment for male infertility, researchers and clinicians may not be able to fully understand its potential benefit for patients, in the context of previously published research. A core outcome set allows researchers to measure a consistent set of clinical endpoints.

By developing a core outcome set for male infertility research, we hope to harmonize the outcomes collected and published in future research. We hope this will better inform clinical decision-making for healthcare professionals and improve the care patients receive.

## Introduction

Infertility is defined as the failure to achieve a clinical pregnancy following 12 months of regular unprotected sexual intercourse (Zegers-Hochschild *et al.*, 2009). Male factor infertility affects 18 million men globally and is recognized by the World Health Organization (WHO) as a critical public health issue (Mascarenhas *et al.*, 2012; Winters and Walsh, 2014; Agarwal *et al.*, 2015; Lotti and Maggi, 2018). Identifiable and therefore potentially modifiable causes of male factor infertility include congenital (genetic), acquired, idiopathic and many other causes (Kovac and Lamb, 2014; Tüttelmann *et al.*, 2018; European Association of Urology, 2019). Despite extensive investigations, most cases of male infertility remain unexplained (Turner *et al.*, 2020). The exploration of factors that impair male fertility is growing and has resulted in randomized trials investigating a wide range of potential interventions. Factors that may impair male fertility include occupational risks, exposure to reproductive toxicants, chemotherapy and radiation therapy, heat exposure, manual work, lifestyle factors such as tight underwear, poor nutrition, genital trauma, genetic traits, testicular maldescent, infection and iatrogenic causes (Cherry *et al.*, 2008; Chung and Brock, 2011; Povey *et al.*, 2012; Cherry *et al.*, 2014; Punab *et al.*, 2016; Schuppe *et al.*, 2017; Mackenzie and Gellatly, 2019; Skoracka *et al.*, 2020; Zhang *et al.*, 2020; Hallast *et al.*, 2021).

The Priority Setting Partnership for Infertility, involving 179 healthcare professionals, 153 patients and 56 others, from 40 countries, has co-produced a research agenda for male infertility (Table I) (Duffy *et al.*, 2020b, 2021a). The majority of these research priorities will need to be addressed within a randomized controlled trial (RCT) setting. When appropriately designed, conducted and reported, RCTs can generate robust evidence (Hariton and Locascio, 2018). Although an individual RCT is useful, pooling data across multiple RCTs provide the best evidence base to inform clinical decision-making (Ahn and Kang, 2018). In order to pool data across several studies, homogeneous outcomes and outcome definitions must be used. This has led to considerable attention being paid to standardizing randomized trial methods and reporting. This includes the Harbin Consensus, which developed an extension, termed the ‘Consolidated Standards of Reporting Trials’ (CONSORT) known as the ‘Improving the Reporting of randomized trials of Infertility Treatment’ (IMPRINT) statement (Schulz *et al.*, 2010; Harbin Consensus Conference Workshop Group *et al.*, 2014a,b). However, the selection, collection and reporting of outcomes and outcome measures have been neglected.

**Table I:** Top 10 research priorities for male infertility.

Top 10 consensus driven research priorities for male infertility
Are sperm tests other than bulk parameters useful in evaluating male fertility? If so, which? What is the emotional and psychological impact of male infertility? Can addressing it improve outcomes? Do environmental factors cause male infertility? If so, which? Does treating specific causes of male infertility improve outcomes? Can we improve surgical sperm extraction outcomes by using endocrine stimulation protocols? What modifiable risk factors cause male infertility? Does treating modifiable risk factors improve outcomes? What co-morbidities are associated with infertility? Does treating co-morbidities improve outcomes? Are nutraceuticals useful in improving male reproductive potential? If so, which?

The Priority Setting Partnership for Infertility, involving 179 healthcare professionals, 153 patients and 56 others including scientists, researchers and methodologists from 40 countries, co-produced a research agenda for male infertility. Ten research priorities were identified.

Published male infertility research has reported diverse outcomes and outcome measures. A systematic review in preparation for this protocol reviewed the 100 largest RCTs evaluating potential treatments for male infertility (Rimmer *et al.*, 2022). Live birth was reported as the primary outcome in only four RCTs and as a secondary outcome in a further eight RCTs. Clinical or biochemical pregnancy was reported by 51 RCTs. Semen parameters were reported by 75 RCTs. Fifty-seven RCTs used the WHO reference standards and a single RCT measured strict criteria, frequently referred to as Kruger strict criteria (Menkveld *et al.*, 1990; Franken *et al.*, 2000; Ketabchi *et al.*, 2018). The remaining RCTs did not define how semen parameters were measured or the quality control standards used to carry out this analysis (Björndahl *et al.*, 2016).

Inconsistent outcome selection, measurement and reporting can be addressed and overcome by developing, disseminating and implementing core outcome sets. A core outcome set represents a minimum data set of outcomes developed using robust consensus science methods engaging diverse stakeholders including healthcare professionals, allied healthcare professionals, scientists, researchers and people with fertility problems. Core outcomes should be routinely utilized by researchers, collected in a standardized manner and reported consistently in the final publication (Williamson *et al.*, 2017; Duffy *et al.*, 2017a,b).

**Figure 1.**
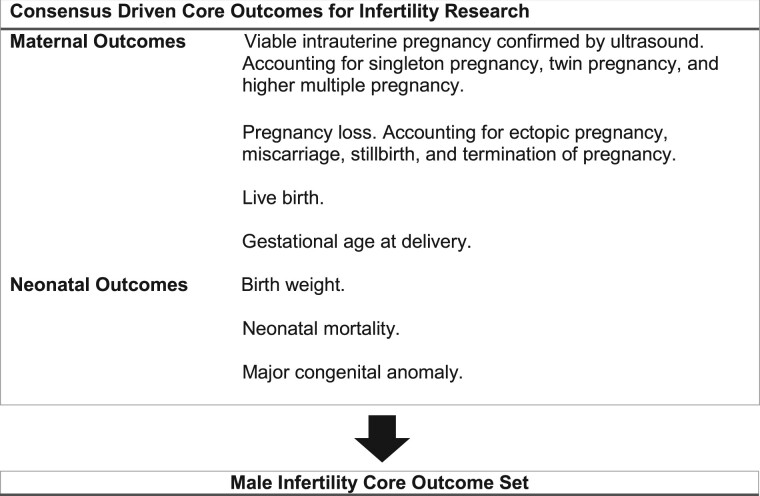
**Core outcome set for infertility research.** A core outcome set for general infertility research has been developed by The Core Outcome Measures for Infertility Trials initiative, which primarily focuses on female infertility. However, the challenges to be addressed in male infertility differ: the nature of male infertility trials means they have up to three potential participants, namely a male and female participant and their offspring, all with potential outcomes to be reported. To address this challenge, the development of a unique male infertility core outcome set is required.

The Core Outcome Measure for Infertility Trials initiative (COMMIT) is an international collaboration committed to improving outcome selection, collection and reporting across fertility research. A core outcome set has been developed for general infertility research, which primarily focuses on female infertility; however, the challenges to be addressed in male infertility are different (Duffy *et al.*, 2020c, 2021b). The nature of male infertility trials means they have up to three potential participants, a male and female participant and their offspring, all with potential outcomes to be reported. To address this challenge, the development of a unique core outcome set relevant to male infertility research is required (Fig. 1) (Duffy *et al.*, 2020c, 2021b).

We aim to produce, disseminate and implement a core outcome set for future male infertility research assessing the efficacy of new interventions to improve the quality of evidence produced through RCTs.

## Materials and methods

### Steering group

**Figure 2.**
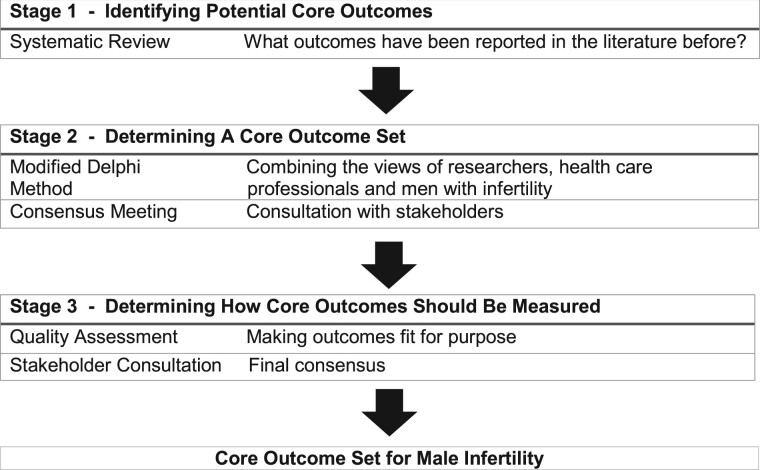
**Developing a core outcome set for male infertility trials.** An international steering group, including healthcare professionals, allied healthcare professionals, healthcare scientists, researchers and people with fertility problems, has been formed to guide the development of a male infertility core outcome set. Members of the steering group represent various disciplines, geographical areas and expertise.

An international steering group, including healthcare professionals, allied healthcare professionals, healthcare scientists, researchers and people with fertility problems, has been formed to guide the development of this core outcome set (Fig. 2). Members of the steering group represent various disciplines, geographical areas and expertise.

### Prospective registration

This study has been registered prospectively with the Core Outcome Measures in Effectiveness Trials (COMET) initiative; the registration number is 1586 and is available online (www.comet-initiative.org/Studies/Details/1586).

The study methods have been informed by reviewing previous core outcome sets in women’s and newborn health (Williamson *et al.*, 2012; Khalil *et al.*, 2017; Whitehouse *et al.*, 2017; Duffy *et al.*, 2018; Al Wattar *et al.*, 2020; Duffy *et al.*, 2020a; Doumouchtsis *et al.*, 2020; Ghai *et al.*, 2020; Kim *et al.*, 2020).

### Step 1: Identification of potential core outcomes (what outcomes have been reported before?)

The Cochrane Central Register of Controlled Trials (CENTRAL) is a bibliographical database containing randomized trial reports identified by searching other bibliographical databases, including EMBASE, Medline and PubMed. We will search CENTRAL to identify RCTs evaluating potential treatments for male infertility. The screening of title and abstracts will be performed in duplicate and disagreements will be resolved by discussion with a third reviewer. No language restrictions will be applied, and translations will be sought for non-English language reports. Full-text reports will be reviewed for eligible trials. Data will be extracted in duplicate using a standardized and piloted data extraction proforma recording study characteristics and primary and secondary outcome reporting. Disagreements will be resolved by discussion with a third reviewer. Individual outcomes will be entered into the outcome inventory, which will be incorporated into a modified Delphi method (Dalkey and Helmer, 1963).

### Step 2: Determining core outcomes (combining the views of healthcare professionals, researchers and people with fertility problems)

The modified Delphi method enables key stakeholders to participate in a process that assesses the extent of agreement (consensus measurement) and resolves disagreement (consensus development) (Williamson *et al.*, 2017). Key stakeholders, including healthcare professionals, allied healthcare professionals, scientists, researchers and people with fertility problems, will be invited to participate. Although some Delphi’s have focused on expert opinions to reach a consensus, the development of previous core outcome sets as part of the COMMIT initiative has included patients in all aspects of the Delphi process. Patients play an active role in ranking the importance of proposed outcomes as well as participating in discussion on how they should be measured and defined.

No robust methodology is available to calculate the required sample size for a Delphi consensus; however, we aim to recruit a minimum of 16 participants from each stakeholder group based on previous work in the field of infertility (Duffy *et al.*, 2020c, 2021b).

During the first round of the Delphi consensus, participants will be asked to provide their demographic details and be allocated a unique identifying number to ensure future anonymity. Proposed core outcomes will be presented within each domain. Participants will be invited to score individual outcomes using a nine-point scale from one (labelled no importance for decision-making) to nine (labelled critical importance for decision-making) (Guyatt *et al.*, 2011; Williamson *et al.*, 2017). This scale was devised by the Grading of Recommendations Assessment, Development and Evaluation (GRADE) working group to facilitate the ranking of outcomes according to their importance and has been adopted widely by core outcome set developers (Guyatt *et al.*, 2011; Williamson *et al.*, 2012). Participants will be presented with the opportunity to add additional outcomes before completing the survey.

All outcomes will be carried forward from round one to round two. Participant’s scores will be calculated for each outcome, and the results obtained for each outcome will be represented in a histogram based on the stakeholder groups responses. The steering group will consider additional outcomes proposed by stakeholders. Those included will be presented with the initial round one outcomes and circulated in round two of the Delphi consensus.

During round two of the survey, participants will receive the summary scores from all participants in round one. Participants will be invited to reflect on the summarized stakeholder group feedback, re-score round one outcomes, and score the additional outcomes suggested by participants in round one.

On completion of round two, a consensus definition would be identified when >70% of participants in each stakeholder group scored the outcome ‘critical for decision-making’ (score seven to nine) and <15% of participants in each stakeholder group scored the outcome ‘of limited importance for decision-making’ (score one to three).

Although the modified Delphi process allows a multinational, multiprofessional consensus to be reached, there are limitations to this approach. A lack of robust methods to determine the number of participants required means a sample size calculation cannot be undertaken: instead numbers of participants in previous studies are used to guide researchers planning future studies. Answering large numbers of questions in multiple rounds of the Delphi process can lead to participant fatigue and attrition throughout subsequent rounds. The global reach of a modified Delphi delivered online means non-English speaking participants may not engage as effectively with some aspects of the Delphi as native English speakers.

On completion of the modified Delphi, a consensus development workshop will be conducted using a modified Nominal Group Technique. Healthcare professionals, researchers and men with infertility who completed both rounds of the Delphi survey will be invited to participate in the consensus development meeting. The modified Nominal Group Technique does not depend on statistical power and there is no robust method for calculating the required number of participants (Murphy *et al.*, 1998). The study will aim to recruit a minimum of 10–15 participants, ensuring representation from healthcare professionals, researchers and men with lived experience of infertility. Consensus development meetings of 10–15 participants have been used to reach an agreement in other settings and will be used in this consensus development meeting (Murphy *et al.*, 1998; Duffy *et al.*, 2020c, 2021b).

Prior to the meeting, participants will be asked to provide demographic details and commit to active participation. All consensus outcomes will be entered into the process. Participants can enter additional outcomes which do not reach the consensus threshold upon request. Outcomes will be divided into three provisional categories: outcomes to be considered for inclusion in the final core outcome set; outcomes where no consensus was reached; and outcomes that will not be considered for inclusion in the final core outcome set.

Participants will be invited to discuss the ordering of the outcomes within each category, considering contextual information, including the relative importance of individual outcomes, feasibility to collect the outcome data in future trials and the availability of suitable definitions and measurement instruments. They will be encouraged to reformulate outcomes to improve clarity or comprehension. The discussion among participants will focus on ranking outcomes to be considered for inclusion in the final core outcome set and the outcomes where no consensus existed. During the discussion, outcomes can be moved between categories. Finally, the core outcomes will be agreed upon.

### Step 3: Identification and standardization of core outcome measures (ensuring outcome measures are fit for purpose)

Once core outcomes have been agreed upon by the Delphi consensus, we will determine how these outcomes should be defined and measured. A systematic review of clinical national and international guidelines, Cochrane reviews and randomized trials will be undertaken to identify potential definitions, from inception until July 2021. Development initiatives relevant to infertility research will be identified by systematically reviewing the COMET initiative register. In addition, a systematic review of national and international male fertility guidelines as well as Cochrane reviews will be undertaken to source definitions as well as reviewing definitions used in the 100 largest RCTs published in male fertility over the past 10 years. Combining these sources, an inventory of potential definitions will be developed. These definitions will be entered into a consensus development conference involving stakeholders from each group, as previously described (Ferguson, 1996). This method was developed to incorporate judicial decision-making, scientific conferences and the town hall meeting. During the consensus development, participants hear evidence on which they will deliberate and ask questions as the evidence is presented.

Healthcare professionals, allied healthcare professionals, scientists, researchers and men with lived experience of fertility problems will be invited to participate in the consensus development workshop. The number of individuals to include in the consensus development study does not depend on statistical power but requires representation from each stakeholder group. Previous consensus group meetings to establish core outcomes and core outcome definitions have had 17–41 participants but can be as few as 10 participants (World Health Organization, 2014; Duffy *et al.*, 2020b,c,d, 2021a,b,c).

Potential measurement instruments will be inventoried against national and international guidelines, Cochrane reviews and randomized trials. The quality of these instruments will be assessed using the COMET initiative and the Consensus-Based Standards for the Selection of Health Measurements instruments (COSMIN) initiative quality assessment framework (Prinsen *et al.*, 2016).

### Ethical review

The National Research Ethics Service, UK, advised that the study does not require formal review.

## Discussion

The COMMIT initiative has developed a strategic plan in consultation with a broad range of stakeholders across the research pipeline to utilize available enablers to secure the routine selection, collection and reporting of core outcomes across future fertility research (Devall *et al.*, 2020). We are now developing a core outcome set for future male infertility research.

To reduce research waste, funding bodies are increasingly advocating using core outcome sets within the work they fund (Ioannidis *et al.*, 2014). It is deemed good practice for researchers planning RCTs to follow the Standard Protocol Items: Recommendations for Interventional Trials (SPIRIT) statement, which outlines the scientific, ethical and administrative elements incorporated in a clinical trial protocol (Chan *et al.*, 2013). This statement specifically recommends that researchers collect and report core outcomes.

This study will set out to develop a core outcome set for male infertility research. However, during this work, we systematically reviewed outcome reporting in the 100 largest randomized trials in male infertility in the past 10 years. We identified that when trials did report the same outcome, different definitions were often used for these outcomes, e.g. semen analysis, pregnancy rate or live birth. We did not evaluate how these trials undertook these assessment, for example how semen analysis was conducted or if this was in an International Standards Organization accredited laboratory (Björndahl *et al.*, 2016). The COMMIT collaborative has recently developed standardized definitions for general infertility research, much of which is focused on female infertility (Duffy *et al.*, 2020d, 2021c). These definitions were developed using formal consensus development methods for individual core outcomes; however, a core outcome set specifically for male infertility research is required. This additional congruence across future male infertility trials should ensure secondary research can be undertaken prospectively, efficiently and harmoniously (Duffy *et al.*, 2020d, 2021c). This standardization will be supported by developing a freely available electronic case report form and data repository (COMMIT-Collection), which future researchers will be encouraged to use for data collection.

The Core Outcome in Women’s Health (CROWN) initiative, supported by 84 specialty journals, including the *Cochrane Gynaecology and Fertility Group*, *Fertility and Sterility* and *Human Reproduction*, has resolved to implement this male infertility core outcome set (Khan, 2016). CROWN initiative journals will advise researchers to collect and report the core outcome set for male infertility within-trial reports and offer conclusions based on these outcomes. Where core outcome sets have not been collected, the researchers will be asked to report this and its implications for their findings. The COMMIT initiative is currently developing reporting tools and templates to assist researchers in clearly reporting core outcomes within their manuscripts (COMMIT-Reporting).

The *Cochrane Gynaecology and Fertility Group* have published over 100 systematic reviews evaluating potential treatments for infertility and has committed to implementing the core outcome set for male infertility when new and updated reviews are being prepared. Secondary research, including pairwise meta-analyses, individual participant data meta-analyses and network meta-analyses, will be more influential when male infertility trials routinely collect and report core outcomes.

The COMMIT initiative has committed to undertaking further research to assess the uptake and implementation of the core outcome set for male infertility (COMMIT-Implementation). Objectively demonstrating the uptake of the core outcome set for infertility is important to quantify its contribution to improving the value of future research. Assessing the uptake of the core outcome set will be undertaken by examining registry records, published protocols, RCT and systematic reviews, and undertaking a citation analysis. Further research is planned to examine and understand why researchers do, and do not, implement the core outcome set for male infertility. By identifying the perceived barriers to the utilization of a core outcome set for male infertility trials, strategies informed by implementation science will be developed to limit, and hopefully overcome, this.

## Data Availability

No new data were generated or analysed in support of this research.

## Authors’ roles

M.P.R., R.A.H., J.M.N.D., C.M.F. and C.N. conceived the idea, developed the protocol and wrote the manuscript.

R.A.A., C.L.R.B., K.T.B., Y.B., R.P.B., S.B., L.B., P.B., R.E.B., A.E.P.C., E.C., B.L.C., K.C., M.L.E., C.De.G., D.G.G., R.R.H., V.N.A.H., A.F.H., C.H., J.H.K., M.S.K., S.K., Y.K., J.K., T.K., S.L., T.C.M., R.S.M., K.Mc.E., R.T.M., B.W.M., A.M.M., E.H.Y.N., A.P., A.H.P., S.Du.P., N.R., I.S., P.N.S., M.Sh., M.Śm., V.S., S.K.S., B.C.T., F.T., A.V., M.v.W., M.H.V.-L., L.N.V., A.Y.W., R.W., and A.Z. supported the development of the protocol participated in the preparation and critical appraisal of the manuscript.

All authors approved the final version of the manuscript.

## Funding 

This work has been supported by the Urology Foundation small project award, 2021.

## Conflict of interest

C.L.R.B. is the recipient of a BMGF grant and received consultancy fees from Exscentia and Exceed sperm testing, paid to the University of Dundee and speaking fees or honoraria paid personally by Ferring, Copper Surgical and RBMO. S.B. received royalties from Cambridge University Press, Speaker honoraria for Obstetrical and Gynaecological Society of Singapore, Merk SMART Masterclass and Merk FERRING Forum, paid to the University of Aberdeen. Payment for leadership roles within NHS Grampian, previously paid to self, now paid to University of Aberdeen. An Honorarium is received as Editor in Chief of *Human Reproduction Open*. M.L.E. is an advisor to the companies Hannah and Ro. B.W.M. received an investigator grant from the NHMRC, No: GNT1176437 is a paid consultant for ObsEva and has received research funding from Ferring and Merck. R.R.H. received royalties from Elsevier for a book, consultancy fees from Glyciome, and presentation fees from GryNumber Health and Aytu Bioscience. Aytu Bioscience also funded MiOXYS systems and sensors. Attendance at Fertility 2020 and Roadshow South Africa by Ralf Henkel was funded by LogixX Pharma Ltd. R.R.H. is also Editor in Chief of *Andrologia* and has been an employee of LogixX Pharma Ltd. since 2020. M.S.K. is an associate editor with *Human Reproduction Open*. K.Mc.E. received an honoraria for lectures from Bayer and Pharmasure in 2019 and payment for an ESHRE grant review in 2019. His attendance at ESHRE 2019 and AUA 2019 was sponsored by Pharmasure and Bayer, respectively.
