# Correction to: Protocol for developing a core outcome set for male infertility research: an international consensus development study

**DOI:** 10.1093/hropen/hoaf065

**Published:** 2025-11-02

This is a correction to: Michael P Rimmer, Ruth A Howie, Richard A Anderson, Christopher L R Barratt, Kurt T Barnhart, Yusuf Beebeejaun, Ricardo Pimenta Bertolla, Siladitya Bhattacharya, Lars Björndahl, Pietro Bortoletto, Robert E Brannigan, Astrid E P Cantineau, Ettore Caroppo, Barbara L Collura, Kevin Coward, Michael L Eisenberg, Christian De Geyter, Dimitrios G Goulis, Ralf R Henkel, Vu N A Ho, Alayman F Hussein, Carin Huyser, Jozef H Kadijk, Mohan S Kamath, Shadi Khashaba, Yoshitomo Kobori, Julia Kopeika, Tansu Kucuk, Saturnino Luján, Thabo Christopher Matsaseng, Raj S Mathur, Kevin McEleny, Rod T Mitchell, Ben W Mol, Alfred M Murage, Ernest H Y Ng, Allan Pacey, Antti H Perheentupa, Stefan Du Plessis, Nathalie Rives, Ippokratis Sarris, Peter N Schlegel, Majid Shabbir, Maciej Śmiechowski, Venkatesh Subramanian, Sesh K Sunkara, Basil C Tarlatzis, Frank Tüttelmann, Andy Vail, Madelon van Wely, Mónica H Vazquez-Levin, Lan N Vuong, Alex Y Wang, Rui Wang, Armand Zini, Cindy M Farquhar, Craig Niederberger, James M N Duffy, Protocol for developing a core outcome set for male infertility research: an international consensus development study, *Human Reproduction Open*, Volume 2022, Issue 2, 2022, hoac014, https://doi.org/10.1093/hropen/hoac014.

In the originally published version of the manuscript there was an error in author Basil C Tarlatzis’ last name. This should read: “Tarlatzis” instead of: “Tarlarzis”. The error is outlined only in this notice to preserve the version of record.

